# Nano‐Roughness‐Mediated Macrophage Polarization for Desired Host Immune Response

**DOI:** 10.1002/smsc.202300080

**Published:** 2023-08-13

**Authors:** Panthihage Ruvini L. Dabare, Akash Bachhuka, Jing Yang Quek, Lluis F. Marsal, John Hayball, Krasimir Vasilev

**Affiliations:** ^1^ UniSA STEM University of South Australia Mawson Lakes SA 5095 Australia; ^2^ Department of Electronics, Electric, and Automatic Engineering Rovira I Virgili University (URV) 43007 Tarragona Spain; ^3^ Experimental Therapeutics Laboratory UniSA Clinical and Health Sciences University of South Australia City East Campus Adelaide 5000 Australia; ^4^ College of Medicine and Public Health Flinders University Sturt Road Bedford Park SA 5042 Australia

**Keywords:** foreign body reaction, macrophage polarization, macrophage response, nanotopography, plasma polymerization

## Abstract

Macrophage polarization is a significant event in the host immune response, which can be modulated by modifying the surface of a biomaterial. Previous studies have demonstrated the modulation of macrophage polarization using different surface features; however, none of these studies reflect the effect of surface properties on unstimulated macrophage polarization for a prolonged period. To better understand the impact of surface features, in this work differentiated THP‐1 cells are employed to control macrophage polarization on nano‐rough surfaces for a duration of 7 days. Model nano‐rough substrates are fabricated by immobilizing gold nanoparticles (AuNPs) of predetermined sizes (16, 38, 68 nm) on a 2‐methyl‐2‐oxazoline thin film, followed by tailoring the outermost surface chemistry. All modified surfaces support high levels of cell adhesion and proliferation. Over time, the expression of pro‐inflammatory cytokines decreases, whereas the expression of anti‐inflammatory cytokines increases on all modified surfaces. Similarly, pro‐inflammatory interleukin (IL)‐1β gene expression is downregulated, and anti‐inflammatory IL‐10‐gene expression is upregulated, regardless of the surface roughness. Analysis of cell morphology reveals that the predominant cell type on the modified surfaces exhibits M2 anti‐inflammatory phenotype. Herein, how surface features can modulate macrophage responses over an extended period is highlighted, offering insights for the development of future biomaterial implants.

## Graphical abstract

Four distinct surfaces with precise nanotopography and tailored surface chemistry are fabricated, using 16, 38, and 68 nm AuNPs and plasma‐polymerized 2‐methyl‐2‐oxazoline (pOX) surface chemistry. All modified surfaces, particularly the 38 nm pOX surface, promote macrophage adhesion and proliferation with M2 phenotypic morphology. In this work, the potential of surface nanotopography for modulating macrophage immune responses toward the anti‐inflammatory response is demonstrated.

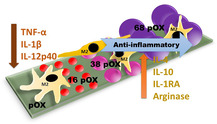

## 1 Introduction

Macrophages play a pivotal role in the foreign body reaction against biomaterial implants by directing the inflammatory response either toward the classical pro‐inflammatory pathway (M1) or the alternative anti‐inflammatory pathway (M2).^[^ ^1^, ^2^ ^]^ M1 macrophages stimulate T helper cell‐1 (Th1)‐type inflammation by expressing pro‐inflammatory cytokines such as interleukin 6 (IL‐6), IL‐1β, and IL‐8. Furthermore, M1 macrophages produce reactive oxygen species, including nitrous oxide (NOS) and nitric oxide (NO) from amino acid metabolism, inducing pro‐inflammatory cascades. In contrast, M2 macrophages induce T helper cell‐2 (Th2)‐type inflammation by secreting cytokines such as IL‐10, IL‐6, and IL‐1RA.^[^ ^3^, ^4^ ^]^ M2 macrophages also produce type I arginase (ArgI), which is required to catalyze polyamine production, synthesize collagen, promote cell proliferation, and tissue remodeling.^[^ ^5^ ^]^ Switching M1 and M2 phenotypes from exhausting phagocytosis events results in the fusion of macrophages to form foreign body giant cells (FBGCs).^[^ ^6^ ^]^ The formation of FBGCs are activated by IL‐4, IL‐13, and granulocyte‐macrophage colony‐stimulating factor, along with fusogens such as CD44, macrophages fusion receptors, dendritic cell‐specific transmembrane protein (DC‐STAMP), mannose receptors, and E‐cadherin, marking the initiation of chronic inflammation.^[^ ^6^, ^7^, ^8^, ^9^, ^10^, ^11^, ^12^, ^13^ ^]^ These FBGCs then direct resulting inflammatory responses toward either pro‐inflammation and material encapsulation or the non‐inflammatory healing process and tissue regeneration.^[^ ^14^ ^]^

Surface features such as chemistry and nanotopography have been known to influence macrophage responses such as adhesion, activation, migration, morphology after adhesion, cytokines secretion (early and delayed secretion), polarization, and FBGC formation.^[^ ^15^, ^16^, ^17^, ^18^, ^19^, ^20^, ^21^, ^22^, ^23^, ^24^ ^]^ Chen et al. reported that surface roughness is the driving factor in controlling the morphology of macrophages, thus regulating phagocytosis.^[^ ^17^ ^]^ Macrophages elongation was observed on topographical gratings of up to 500 nm compared to planar control surfaces, where macrophages retained their native round shape.^[^ ^17^ ^]^ Lee et al. analyzed macrophage responses at time points of 4, 24, and 72 h on nanostructured Ti surfaces modified with divalent cations, namely calcium (Ca) and strontium (Sr).^[^ ^25^ ^]^ Enhanced cellular attachment was reported on pure nanostructured Ti surfaces in the early stages, with no differences observed in the later stages. Moreover, the pure nanostructured Ti surface showed an advanced stage of cellular spreading compared to chemically modified surfaces. However, the Sr‐modified nanostructured Ti surfaces had increased numbers of M2 macrophages indicating nanotopographic surfaces with a suitable bioactive modification were beneficial in bone healing.^[^ ^25^ ^]^ In another study, Christo et al. reported the combinatory effect of nanotopography and surface chemistry on cytokine profiles secreted by macrophages after 7 days.^[^ ^23^ ^]^ Here, nanotopography was fabricated by utilizing different sizes of AuNPs (16, 38, and 68 nm) and tailored outermost surface chemistries were generated by a plasma polymers rich in carboxyl, methyl, and amine functional groups.^[^ ^26^ ^]^ It was noted that the expression of pro‐inflammatory cytokines was downregulated on all surfaces with nanotopography, regardless of surface chemistry.^[^ ^23^ ^]^ Macrophage polarization is a time and microenvironment‐dependent phenomenon that is initiated within 24 h of seeding. In addition, cells can switch between both phenotypes without memory of polarization states.^[^ ^27^ ^]^

In the context of the importance of surface properties on the inflammatory cascade of events upon an implantation of a biomaterial, it is essential to determine the role of nanoscale surface roughness on macrophage responses, including polarization and plasticity, for a prolonged time beyond 24 h.^[^ ^21^, ^28^, ^29^, ^30^ ^]^ Furthermore, a majority of studies utilized lipopolisaccharides (LPS)‐activated macrophages, which in itself triggers significant cytokine secretion. However, LPS activation may mask or misrepresent the real effect of the biomaterial surface in question.^[^ ^16^, ^23^ ^]^ Therefore, using non‐stimulated macrophages maybe represent a more authentic approach to reveal the real impact of surface nano‐roughness on macrophage polarization.

The current study aims to evaluate the effect of nano‐roughness engineered by immobilizing gold nanoparticles (AuNPs) of predetermined sizes (16, 38, and 68 nm) on the response of non‐stimulated macrophages at days (D) 1, D4, and D7 post‐seeding. Plasma‐polymerized 2‐methyl‐2‐oxazoline (pOX) was used as an underlying thin layer to immobilize the AuNPs because of its biocompatibility and capacity to provide covalent nanoparticle binding to the surface.^[^ ^31^, ^32^ ^]^ Furthermore, earlier research has shown that AuNPs surfaces are not harmful to many cell types and that cells thrive on them.^[^ ^33^, ^34^, ^35^, ^36^ ^]^ Differentiated THP‐1 cells were used to study macrophages adhesion, morphology, cytokine release profiles, gene expression, and polarization upon interaction with the nanoengineered surfaces.

## 2 Results and Discussion

The model surfaces used throughout this investigation were fabricated by first applying an underlayer of pOX to cell culture wells, glass coverslips, and other substrates and then immobilizing AuNPs of predetermined sizes. The only variability in the model substrates prepared in this manner is the height of nano‐roughness, achieved by maintaining the equal density of nanoparticles across the surfaces. The outermost surface chemistry was tailored by adding a 5 nm thin layer of pOX plasma polymer.

### 2.1 Surface Characterization

**Figure 1:**
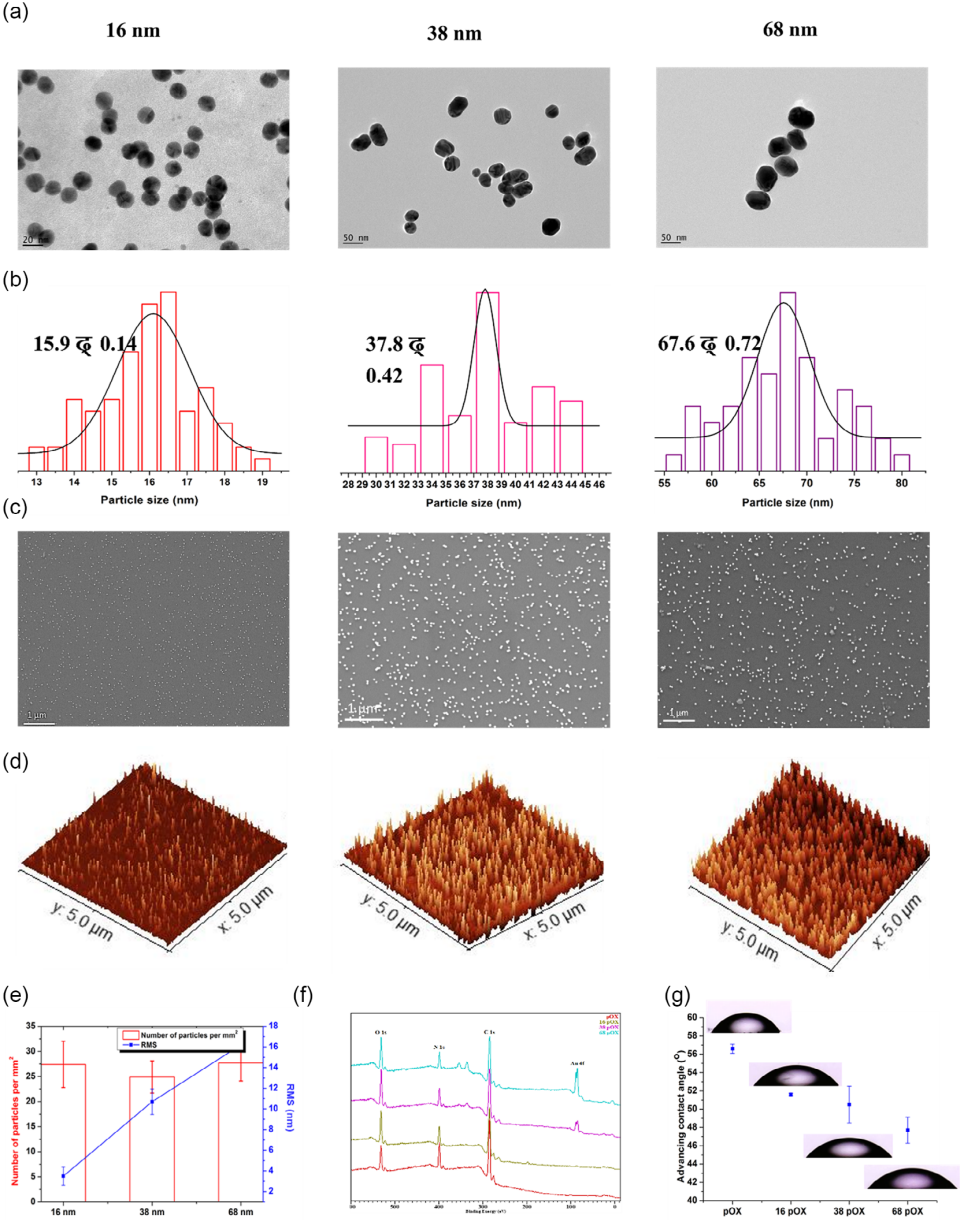
a) Surface characterization of the nanotopography‐ and chemistry‐modified surfaces. Transmission electron microscopy (TEM) images of the nanoparticles used for surface immobilization. b) Particle size distribution obtained from TEM images. c) Scanning electron microscopy (SEM) images showing the distribution of nanoparticles of different sizes on the model surfaces; scale bar: 1 μm. d) 3D atomic force microscopy (3D‐AFM) images of the surfaces after overcoating. e) The number of nanoparticles per unit area and root‐mean‐square (RMS) roughness of different surfaces calculated from AFM images. f) X‐ray photoelectron spectroscopy (XPS) spectra after overcoating. g) Advancing water contact angle.

Transmission electron microscopy (TEM) was utilized to determine the size and shape of AuNPs (16, 38, and 68 nm). The TEM images shown in **Figure** **1**a demonstrate that the nanoparticles have spherical shapes. The mean size distribution of nanoparticles was determined to be 15.9 ± 0.14, 37.8 ± 0.42, and 67.6 ± 0.72 nm, respectively (Figure 1b). The scanning electron microscopy (SEM) images in Figure 1c demonstrate the successful immobilization of nanoparticles on the pOX‐coated surface, their distribution on the surface, and the absence of aggregations. Figure 1d shows 3D atomic force microscope (3D‐AFM) images of the three different sizes of nanoparticles immobilized on the plasma‐polymerized surfaces. These AFM images were used to calculate the number of nanoparticles per unit area and the root‐mean‐square (RMS) roughness of the modified surfaces (Figure 1e). The number of nanoparticles on all modified surfaces was identical, i.e., 26 ± 2, indicating equal particle density. As expected, the RMS increased with the increase in the size of the nanoparticles from 3.4 ± 0.8 (for 16 pOX) to 10.3 ± 1.2 (for 38 pOX) and 16.2 ± 0.45 (for 68 pOX). After immobilization, the modified surfaces have mixed chemistry resultant from the underlying pOX layer and the carboxyl acid groups–capped nanoparticles. To correct this, a thin film of pOX (5 nm) was applied over the immobilized nanoparticles to generate a uniform outermost surface chemistry. X‐ray photoelectron spectroscopy (XPS) was utilized to characterize the surface chemistry before and after overcoating with pOX. XPS survey spectra demonstrate the presence of carbon (C), nitrogen (N), and oxygen (O) on the pOX‐coated surfaces (Figure 1f). An additional gold (Au) peak was obtained after nanoparticle immobilization. The surface atomic concentration of gold increased with the increase in the size of the nanoparticles (Table S1, Supporting Information). After pOX overcoating, a substantial reduction in the amount of gold was observed. However, the gold was still visible as the overcoating was only 5 nm thick while the XPS sampling depth is around 10 nm. Sessile drop contact‐angle measurements demonstrated a decrease in the water contact angle with an increase in nanoparticle size, which is in an agreement with the Wenzel wetting state (Figure 1g).^[^ ^37^ ^]^

These results demonstrate the successful fabrication of surfaces with well‐defined nanotopography and chemistry and validate that roughness height is the only variable parameter. These surfaces were next utilized to study the effect of surface roughness on macrophage behavior and polarization for the time period of up to 7 days.

### 2.2 Cell Adhesion and Cell Morphology

Differentiated THP‐1 (dTHP‐1) cells were seeded on nanotopography‐modified tissue culture well plates (TCP) to determine cell adhesion, cytokines secretion, immunostaining, and gene expression on D1, D4, and D7 of seeding.

**Figure 2:**
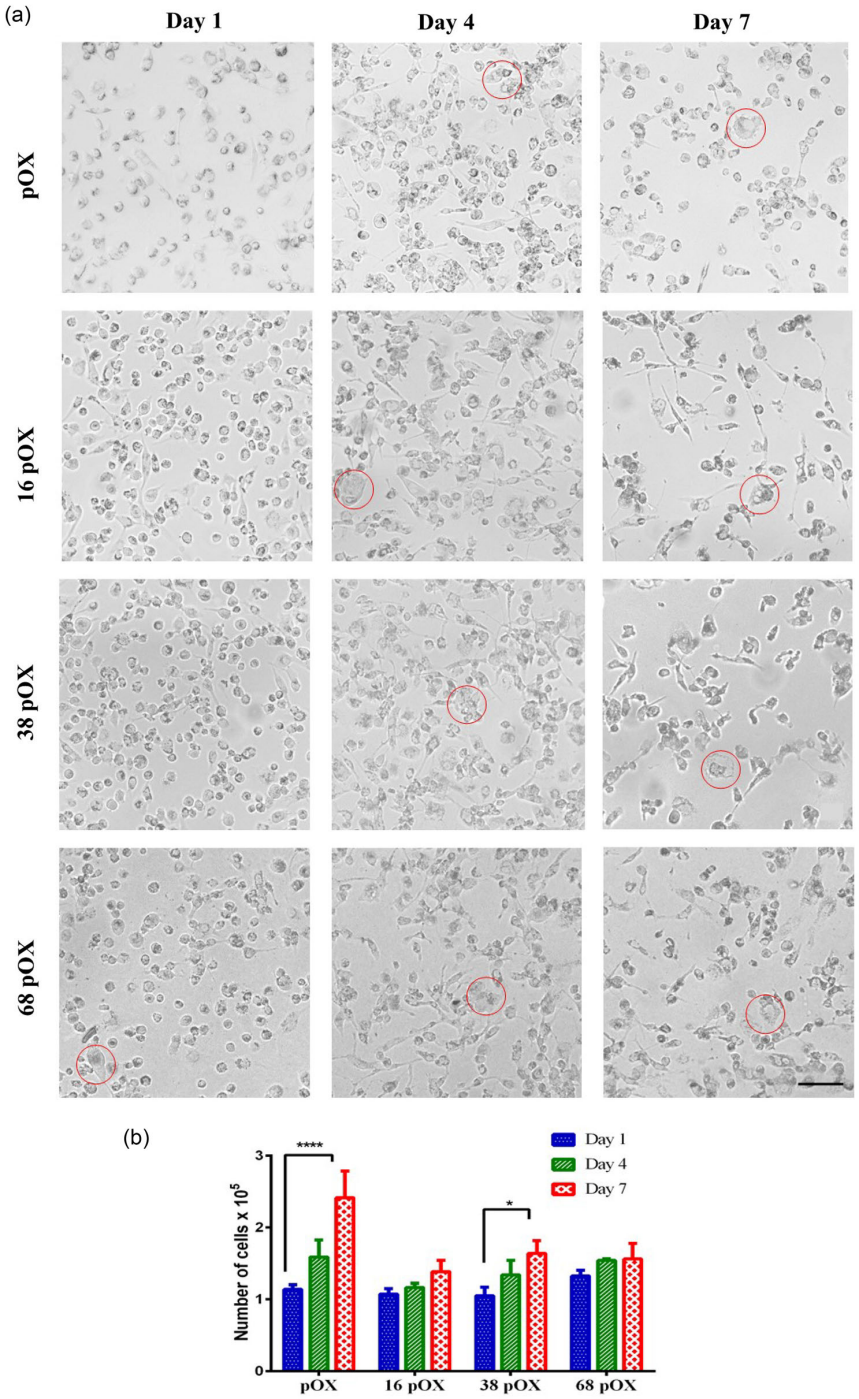
a) Representative bright‐field optical microscopy images of adhered macrophages on the modified surfaces at D1, D4, and D7. The red circles represent large cells and multinucleated giant cells. Significance difference: *p* < 0.05 (*), <0.0001(****), scale bar of images (black color line) = 0.1 mm. b) Number of adhered macrophages on modified surfaces (plasma‐polymerized 2‐methyl‐2‐oxazoline [pOX], 16 pOX, 38 pOX, and 68 pOX) at D1, D4, and D7.

Fluorescence microscopy images obtained for cells adhered to differently modified surfaces are presented in **Figure** **2**. Images at different time points (D1, D4, and D7) revealed cells with different morphology compared to the native macrophages round shape (Figure 2a). The predominated round morphology can be seen on all surfaces at D1 regardless of the surface modification. However, larger circular cells are present on the 68 pOX surfaces compared to more elongated cells on 16 pOX and 38 pOX. Whereas, by D4 and D7, elongated and multinucleated cells (red color circles in Figure 2b) dominated on all surfaces.

The number of cells was calculated from the microscopy images for all modified surfaces (Figure 2b). Cell numbers increased on all surfaces over time (D1 < D4 < D7) due to cellular proliferation. However, compared to D1, the only significant difference in the number of cells was observed on D7 on surfaces coated with pOX only and 38 pOX (Figure 2b). These results demonstrate that cell adhesion may be independent from the surface features (in the range used in this study) in the early days of implantation (D1). This observation is supported by several studies. Chamberlain et al. demonstrated that after 24 h there was no significant change in the number of macrophages adhered on surfaces modified with different sized TiO_2_ nanotube.^[^ ^38^ ^]^ In a similar study, Lee et al. reported no significant difference in macrophage adhesion and proliferation on nanostructured Ti surfaces modified with divalent cations over an extended time duration (3 days).^[^ ^25^ ^]^ However, our study indicates that surface features can control cell adhesion over an extended period (i.e., D7).

**Figure 3:**
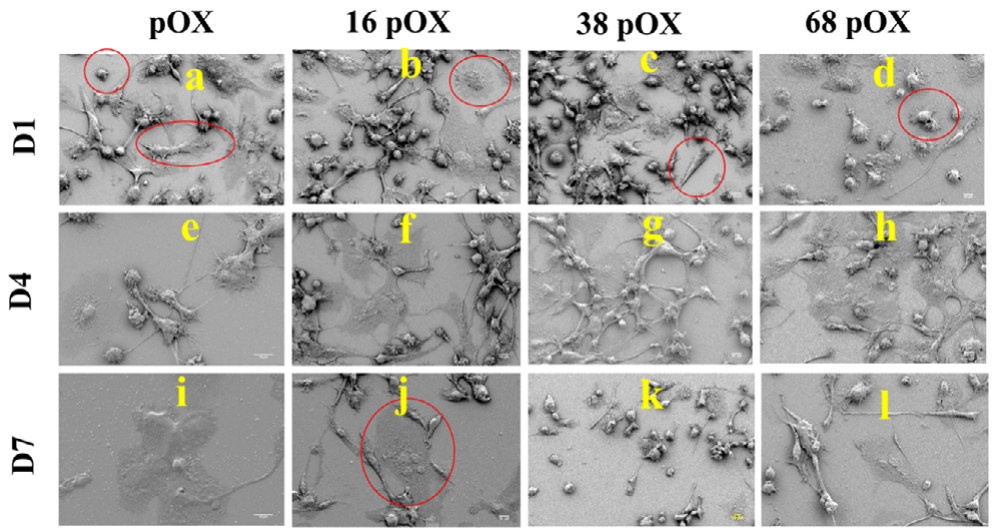
SEM images of macrophages on nanotopographic surfaces at different time points—D1, D4, and D7. The red circles represent cells’ shapes, including round cells without cytoplasm expansion, bipolar spindle cells, pancake‐like cells, round cells with small cytoplasm, and foreign body giant cells (FBGCs). Scale bar = 10 μm.

SEM images of all modified surfaces were acquired to further evaluate cellular morphology and its role in macrophage polarization (**Figure** **3**). The round macrophages are largely unpolarized and unstimulated M0 phenotypes, which have a round shape with limited cytoplasmic extensions.^[^ ^39^ ^]^ Macrophages change shape over time as they polarize into M1 and M2 phenotypes. The morphology of unpolarized and unstimulated macrophages (M0) is small and round, without cytoplasmic extensions or spreading.^[^ ^39^ ^]^ Pro‐inflammatory M1 macrophages display a pancake‐like shape or round structure.^[^ ^40^, ^41^, ^42^ ^]^ In contrast, anti‐inflammatory M2 cells can adapt their shape and represent several morphologies, such as elongated type, round shape, amoeboid type, and bipolar spindle shape.^[^ ^39^, ^40^, ^42^, ^43^ ^]^ The morphology of M1 and M2 was found depended on surface features. While both phenotypes exhibited round shapes on glass, M2 cells display elongated structures on Ti surfaces.^[^ ^41^ ^]^ M0 cells are highly spread on flat bulk metallic glass surfaces, whereas M1 and M2 are more spread, with podia on nanopatterned bulk metallic glass surfaces.^[^ ^42^ ^]^ A distinct change in macrophages morphology was reported on different diameters of nanotube surfaces.^[^ ^38^ ^]^

SEM images demonstrate that macrophages were firmly attached after 24 h incubation and had a round, spindle, amoeboid, and elongated shape morphology. The round macrophages shown in Figure 3 are largely unpolarized and of the unstimulated M0 phenotypes, which have a round shape with limited cytoplasmic extensions. These circular M0 macrophages are inactive or have baseline characteristics. The cytoplasm was extended around the nuclei on the pOX surface (Figure 3a), suggesting the initiation of polarization within 24 h. The morphology of the macrophages on 16 nm pOX comprised round, pancake‐like, spindle shape cells, representing the polarization of cells toward the M1 phenotype (Figure 3b). Cells on 38 nm pOX were stimulated mainly toward the M2 phenotype, as elongated, spindle, and round shape morphology was observed (Figure 3c). Cells were larger than normal round cells on all modified surfaces (Figure 3). Macrophages on 68 nm pOX revealed three morphologies: round shape with minimal cytoplasm expansion, elongated, and pancake‐like structures. Although macrophages commenced polarization toward both M1 and M2, M0 macrophages could still be seen on the 68 nm pOX surfaces (Figure 3d).

At D4, on the pOX surface, macrophage morphology was distinctly altered due to the expansion of the cytoplasm (Figure 3e). There were very few pancake‐like and multinucleated cells among the spindle and elongated cells. Cell‐to‐cell interactions were observed through filopodia (Figure S1, Supporting Information). At D4, the predominant morphology on the 16 nm pOX surface was elongated cells, with a small number of larger cells having highly expanded cytoplasm, a few round cells with minimal cytoplasm expansion, and pancake‐like cells (Figure 3f). The cells did communicate through filopodia and were attached firmly to the surface (Figure S2, Supporting Information). Elongated and bipolar spindle cells were the abundant cell types on 38 pOX at D4, with few FBGCs on the surface (Figure 3g and S3, Supporting Information). The morphology of macrophages at D4 on the 68 nm pOX surface included spindle type, round cells with minimal cytoplasm, pancake‐like structures, and a few FBGCs (Figure 3h and S4, Supporting Information).

At D7, the cells on the pOX surface were highly firmed, and the cell size increased to around 40 μm; the size doubled compared to D1. Cell‐to‐cell interaction was still persistent, as the surface facilitated cell adhesion and communication. Moreover, multinuclei FBGCs with flattened nuclei were present on the surface (Figure 3i and S1, Supporting Information). Compared to macrophages on the pOX surface, at D7, most of the cells on 16 nm pOX maintained mononucleated cells with identical morphologies as at D4. A few FBGCs with flattened structure were present on these surfaces, similar to the pOX surface (Figure 3j). On 38 nm pOX surfaces, the morphology of macrophages included bipolar spindle type, round cells with protruded nuclei, elongated type, and FBGCs, same as that of D4 (Figure 3k and S3, Supporting Information). There were many flattened cells on 68 nm pOX surfaces at D7, along with bipolar spindle cells and amoeboid cells with lengthy filopodia (Figure 3l and S4, Supporting Information).

The existence of round macrophages at later time points, such as D3 or D7, may also represent M1‐polarized cells, which have a round shape with enlarged cytoplasm. As a result, the observed round macrophages in our study include both the M0 and M1 phenotypes, representing various stages and phenotypes of macrophage activation and responsiveness to surface changes.

### 2.3 Macrophage Polarization Based on Cytokine Analysis

The morphology of the macrophages indicated their polarization (M0, M1, and M2) over different time points (D1, D4, and D7) on differently modified surfaces. To validate our qualitative data, cytokine analysis was performed.

**Figure 4:**
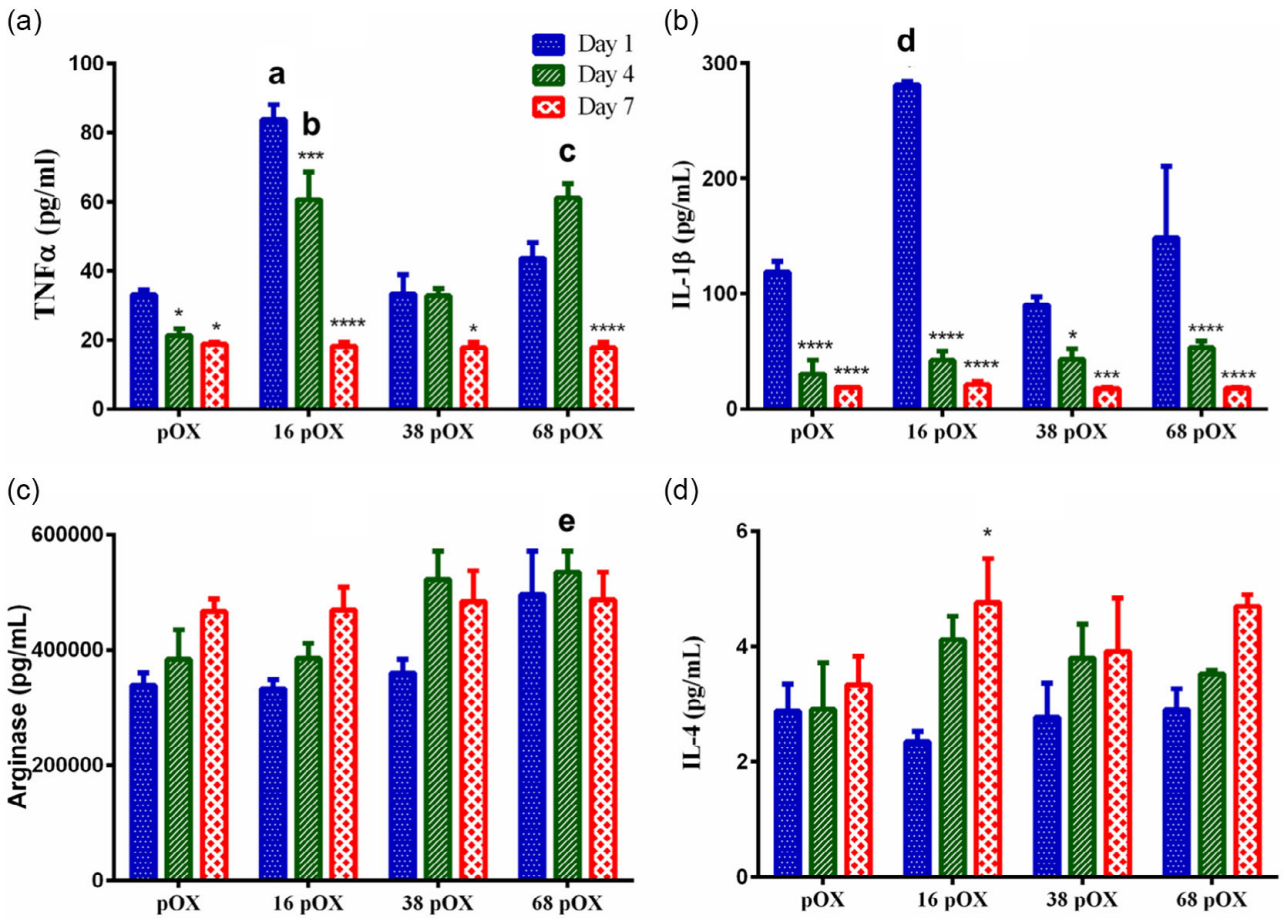
Cytokines secretion profile from macrophages in D1, D4, and D7. a) Pro‐inflammatory TNF‐α. b) Pro‐inflammatory IL‐1β. c) Anti‐inflammatory arginase. d) Anti‐inflammatory IL‐4. Statistical difference = asterisk represents the significance among days, * <0.05, ** <0.01, *** <0.001, and **** <0.0001, simple letters represent the significance among surfaces each day compared to pOX surface; *a* >0.0001, *b* >0.0001, *c* >0.0001 for TNF α, *d* <0.0001 for IL‐1β, and *e* <0.5 for arginase.

The expression of pro‐inflammatory cytokines (tumor necrosis factor alpha (TNF‐α) and IL‐1β) decreased over time on all types of surfaces. The expression of Tumor necrosis factor alpha(TNF)‐α at D1 was higher on the 16 nm pOX surface compared to the pOX surface (*p* < 0.0001). Whereas, no significant difference was observed in the expression of TNF‐α on 38 nm pOX and 68 nm pTumor necrosis factor alpha **4**a). These results align well with the morphology data (Figure 3), suggesting that the polarization on the 16 nm pOX surface is toward the M1 phenotype at D1. At D4, both 16 nm pOX and 68 nm pOX surfaces significantly increased the expression of TNF‐α compared to the smooth pOX surfaces (*p* < 0.0001). On the 68 nm pOX surfaces, TNF‐α secretion increased significantly on D4 compared to D1 (*p* < 0.01) (Figure 3). However, there was an overall reduction in the expression of TNF‐α at D7 regardless of surface modification. The expression of IL‐1β at D1 was like that of TNF‐α, where only 16 nm pOX surfaces showed higher expression than the planar pOX surface (*p* < 0.0001). By D7, the expression of IL‐1β decreased significantly across all surfaces (Figure 4b). The expression of anti‐inflammatory cytokines (arginase and IL‐4) increased over time, although not significant, on all modified surfaces, indicating the polarization of macrophages toward the M2 phenotype. Collectively, the expression of pro‐inflammatory cytokines decreased, whereas the expression of anti‐inflammatory cytokines increased over time on all modified surfaces.

### 2.4 Gene Expression Analysis

**Figure 5:**
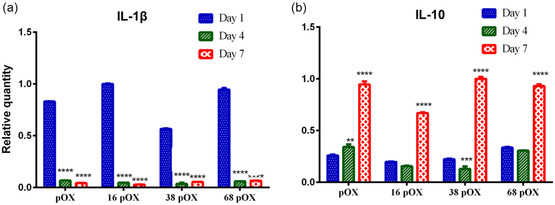
a,b) Gene expression of IL‐1β (a) and IL‐10 (b) on the modified surface at different time points (Day [D]1, D4, and D7); the asterisks represent the significance among days, * <0.05, ** <0.01, *** <0.001, and **** <0.0001.

Gene expression for IL‐1β and IL‐10 was performed to obtain a better insight into the effect of nanotopography on macrophage polarization (**Figure** **5**). The expression of the IL‐1β gene exhibited the same trend as the corresponding cytokine and significantly decreased from D1 to D7. This is a further validation of our findings (Figure 3 and 4) that there is a reduction in the number of pro‐inflammatory macrophages (M1) on the surfaces over time, regardless of modification. At D1, there was a reduction of IL‐1β expression on 38 pOX (*p* < 0.0001), while 16 pOX and 68 pOX (*p* < 0.0001) showed an upregulation compared to pOX (Figure 5a). Chen et al. reported an upregulation in the expression of IL‐1β gene level of LPS‐activated RAW macrophages on surfaces with nanotopography compared to pOX after 6 h of activation.^[^ ^44^ ^]^ At D4 and D7, the expression of the IL‐1β gene reduced significantly on all surfaces compared to D1 (*p* < 0.0001) (Figure 5a).

IL‐10 is a potent anti‐inflammatory cytokine, which indirectly inhibits secretion of pro‐inflammatory cytokines (IL‐1β, IL‐6, and TNF‐α) from monocytes and macrophages by inhibiting the NFκB signaling pathway.^[^ ^45^, ^46^ ^]^ In our study, gene expression of IL‐10 increased over time on all surfaces regardless of surface modification (Figure 5b). The upregulation of IL‐10 correlates with reduced secretion of TNF‐α and IL‐1β as IL‐10 may inhibit their secretion. On D1, the gene expression of IL‐10 was downregulated on 16 nm pOX surfaces (*p* < 0.05) and upregulated on 68 nm pOX surfaces (*p* < 0.01) compared to pOX alone. The gene expression of IL‐10 was downregulated on 16 nm pOX and 38 nm pOX surfaces compared to pOX (*p* < 0.0001) at D4. However, gene expression of the IL‐10 increased at D7 regardless of surface modification, while expression was downregulated on 16 nm pOX surfaces compared to pOX alone (*p* < 0.0001). The upregulation of IL‐10 correlated with the pro‐inflammatory cytokine secretions, which decreased over the time.

The fate of a biomaterial after implantation could lead to either of the two pathways: a) pro‐inflammatory reaction and rejection from the host, or b) upregulation of anti‐inflammatory factors expression and successful wound healing. To gain insight and control into these pathways, the rational engineering of nanotopography and chemistry on the surface of a biomaterial can offer a cutting‐edge platform. In this article, surface nanotopography with height of 16, 38, and 68 nm were fabricated to understand the effect of nanoscale surface roughness on the polarization of unstimulated macrophage for up to 7 days. The results demonstrated that cell adhesion is independent of surface features (in the range used in this paper) in the early days of implantation (D1). Furthermore, all modified surfaces facilitate macrophage proliferation and polarization regardless of the scale of surface nanotopography. The 38 nm pOX surface shows the most favorable immune response as it leads to an increase in the secretion of anti‐inflammatory cytokines while decreasing proinflammatory cytokines compared to other surfaces. It should be noted that this study was focused on the immune response from unstimulated macrophages, which is important to understand the real effect of surface modification on immune cells response. Most of the published studies so far use LPS‐activated macrophages which may mask the effect of surface modification itself since the overwhelming secretion of cytokines in response to bacterial toxins. The knowledge gained from this study provides useful information for designing surfaces that lead to desired immune responses to implantable biomaterials.

Surface modification techniques outlined in this study, such as plasma modification and AuNP incorporation, can generate nanotopography and introduce chemical modifications to various biomaterial surfaces including those made of titanium (Ti), a material often utilized in implantable devices. Several studies have utilized nanoengineered Ti implants for immunomodulation.^[^ ^47^, ^48^ ^]^ However, due to transferability across many different materials, plasma surface modification in combination with immobilized AuNPs offers an exciting option for achieving desired immunological response. While our study gives useful insights into the effects of different surface nanotopography on cellular function, more research is needed to investigate the compatibility and potential synergistic effects of these approaches in the context of Ti‐based implants. The use of nanoengineered Ti implants for immunomodulation, as well as the potential benefits of combining different surface modification strategies, warrant further investigation to fully understand their implications and establish their effectiveness in improving immunological response to Ti implants.

## 3 Conclusions

We have fabricated four different surfaces with well‐defined surface nanotopography and tailored outermost surface chemistry. AuNPs with a diameter of 16, 38, and 68 nm were used to generate model and well‐characterized surface roughness. After immobilizing AuNPs, the surfaces were overcoated with a thin film of pOX which allowed for achieving uniform outermost surface chemistry while preserving the scale of surface roughness. The macrophages were seeded on surfaces, and their adhesion, morphology, cytokines secretion, and gene expression were characterized at D1, D4, and D7 post‐seeding. All modified surfaces supported macrophages attachment and proliferation, and morphology consistent with the M2 phenotype. Moreover, the expression of pro‐inflammatory cytokines was reduced with time on all modified surfaces, while the secretion of anti‐inflammatory cytokines increased, most significantly on the 38 nm pOX surface. Consistently, gene expression of IL‐1β was downregulated, and IL‐10 expression was upregulated with time. Collectively, the results indicate that pOX and the scale of surface nanotopography used in this study could provide a promising platform for modulating immune responses of macrophages toward the wound healing M2 phenotype. This study provides important insights as to how to modify biomaterial surfaces via tailored nanotopography and chemistry, to achieve beneficial immune responses.

## 4 Experimental Section

4.1

4.1.1

##### Materials

pOX, gold (III) chloride trihydrate, mercaptosuccinic acid, trisodium citrate, Roswell Park Memorial Institute (RPMI) 1640 media, and phorbol 12‐myristate 13‐acetate (PMA) were purchased from Sigma–Aldrich (Merck group). Sodium hydroxide was obtained from ChemSupply, Australia. penicillin, streptomycin, fetal calf serum (FCS), and trypsin were purchased from Thermo Fischer Scientific. THP‐1 cells were purchased from CellBank Australia.

##### AuNPs Synthesis

AuNPs were synthesized using citrate reduction of gold (III) chloride in a controlled reflux system.^[^ ^32^ ^]^ Briefly, gold (III) chloride (50 μL of 100 mg mL^−1^) was added into Milli‐Q water (50 mL), and the solution was brought to boiling with stirring at 1300 rpm. Then, sodium citrate (10 mg mL^−1^) was added to the solution to reduce gold (III). The amount of added sodium citrate was chosen based on the size of AuNPs. To obtain 16 nm of AuNPs, 1 mL of sodium citrate solution was added. Similarly, 0.5 and 0.3 mL of sodium citrate were added to the boiling gold solution to obtain 38 and 68 nm AuNPs, respectively. The system was boiled for another 20 min to facilitate the formation of nanoparticles. After 20 min, heating was stopped, and the system was stirred at a constant rate until cool. Mercaptosuccinic acid (1.5 mg mL^−1^) and sodium hydroxide (0.81 mg mL^−1^) were added to the gold colloidal solution for the chemical encapsulation of gold NPs with carboxylic acid functional groups.^[^ ^49^ ^]^

##### Plasma Polymerization

pOX plasma polymer layer of 20 nm thickness was deposited on various substrates, including TCPs (Corning), 13 mm glass coverslips (ProSciTech, Australia), and silicon wafers (M.M.R.C. Ltd, Australia) in a custom‐built plasma reactor equipped with 13.56 MHz plasma generator.^[^ ^24^, ^31^, ^50^ ^]^ Substrates were cleaned with acetone and ethanol, followed by drying with N_2_ flow before placing into the plasma reactor. Substrates were further cleaned with air plasma at 1.1 × 10^−1^ mbar pressure and 50 W radio‐frequency power for 5 min. Then, the monomer (pOX) was introduced into the chamber, and plasma deposition was performed at 8 × 10^−2^ mbar pressure and 50 W power for 2 min.

##### AuNPs Immobilization

Surfaces with an equal density of AuNPs were fabricated according to the previously optimized method.^[^ ^32^, ^49^ ^]^ Briefly, the concentration, time of immobilization, and pH were adjusted according to the size of AuNPs to achieve an equal number of nanoparticles on the polymer‐coated surface. The pH of the AuNP solution was maintained at 4, and immobilization was carried out at ambient temperature. pOX‐coated surfaces were incubated for 1 h and 10 min in a 1:10 ratio diluted solution of 16 nm AuNPs. The immobilization time for 38 and 68 nm AuNPs was kept to 1 h 20 min and 5 h, respectively, no dilution was used for AuNPs of these sizes. After immobilization, surfaces were washed with Milli‐Q water to remove any unbound nanoparticles and dried with N_2_ flow.

##### Surface Overcoating to Tailor the Outermost Surface Chemistry

To prepare uniform outermost surface chemistry a thin film (5 nm) of pOX was deposited using 8 × 10^−2^ mbar pressure and 50 W power for 25 s.

##### Surface Characterization

The size and morphology of AuNPs were determined using TEM. The nanoparticle suspension was deposited on carbon‐coated TEM grids and dried overnight. The TEM (JEOL‐2100F‐HR) was conducted at an accelerating voltage of 80 kV. Image J was utilized to process images to obtain the nanoparticle size distribution. The scale bar for the images was 20, 50, and 100 nm, respectively.

##### Nanotopography

Nanotopography was characterized by AFM. NT‐MDT NTEGRA scanning probe microscope (SPM) AFM with a gold‐coated silicon nitride cantilever (NT‐MDT, NSG03) was used in non‐contact mode to obtain images. Resonance frequencies between 65 and 100 kHz with an oscillation amplitude of 10 nm at a scan rate of 0.5 Hz were used. Images were analyzed using Gwyddion software.^[^ ^51^ ^]^

##### Chemical Analysis of Surfaces

Surface chemistry of the modified silicon wafers was evaluated using Kratos Axis Ultra XPS (Kratos Analytical, UK). XPS equipped with a monochromatic Al source was operated at 15 keV and 15 mA. Survey spectra of pOX and AuNPs modified surfaces were obtained over an energy range of 0–1100 eV in 0.5 eV steps using 160 eV pass energy. The XPS data was analyzed using Casa XPS software by calibrating the binding energies of all elements to the binding energy of C1*s* peak at 285.0 eV.^[^ ^52^ ^]^ All chemical analysis was performed on 2 spots in triplicates of triplicates.

##### Wettability

Wettability of nanotopographic surfaces was measured using RD‐SDM02 contact‐angle‐measuring equipment. Advancing water contact angle (angle between the interface of the solid/liquid and liquid/gas) of a water drop on the modified surface was captured using a web camera. These captured images were analyzed using ImageJ software. All chemical analysis was performed on two spots in triplicates of triplicates.

##### Cell Culture

THP‐1 cells were derived from the peripheral blood of acute monocytic leukemic patients.^[^ ^53^ ^]^ These cells were cultured in RPMI medium supplemented with 10% FCS, 1% v/v penicillin, and streptomycin in an incubator set at 37 °C with 5% CO_2_. THP‐1 cells were differentiated into macrophages (dTHP‐1 cells) using 100 ng mL^−1^ PMA for 48 h in T75 flasks. Then, they were replenished and incubated for another 24 h with PMA‐free fresh media. Adhered dTHP‐1 cells were detached from the flask using trypsin in ethylenediaminetetraacetic acid (EDTA) media (0.25%, Life Technologies, Australia).

##### Cell Adhesion and Size

dTHP‐1 cells (5 × 10^5^ cells/well) were seeded and incubated at 37 °C, and 5% CO_2_ in nanotopography‐modified TCPs (12 well). Three wells for each surface modification were used and the experiment was repeated three times. Cell adhesion was measured on D1, D4, and D7 using two different methods. One method was to visualize the detached cells from the surface using an automatic cell counter (Luna, Logos biosystems) after staining them with trypan blue. The second method was imaging using a bright film fluorescence microscope (Olympus IX83) after fixing cells with 4% paraformaldehyde for 15 min. The magnification used for each image was 0.1 mm. The cell counter automatically provides the number of cells after feeding trypan blue–stained cells in the cell‐counting slide. Images obtained from a fluorescence microscope were analyzed using Image J software to determine the number of cells. Cells were replenished on the same day of cell counting.

##### Cell Morphology

Cell morphology was determined for each time point by culturing cells on surface‐modified glass coverslips. Three coverslips for each surface modification were used and the experiment was repeated three times. dTHP‐1 cells adhered to modified surfaces were washed with phosphate buffer saline (PBS, Invitrogen) and were then fixed overnight with 4% paraformaldehyde in PBS containing 4% sucrose, followed by PBS washing. These samples were then dehydrated by immersing them for 5 min (×2) in an ethanol solution gradient (50%, 70%, and 100%). After dehydration, samples were chemically dried in (1:1) ethanol: hexamethyldisilazane (HMDS, Sigma‐Aldrich) for 30 min, followed by 2 h in 100% HMDS. Finally, samples were mounted on aluminum tubs to sputter‐coat with 2 nm platinum using agar high‐resolution sputter coater. The samples were imaged using scanning electron microscopy (SEM, Zeiss Merlin field‐emission gun (FEG) SEM with silicon drift detector (SDD) energy‐dispersive spectroscopy (EDS), Carl Zeiss, Germany). The magnification used for each image was 10 μm.

##### Cytokine Analysis

Macrophages (1 × 10^6^ cells well^−1^) were cultured on nanotopography‐ and chemistry‐modified TCPs (12 wells), and cell supernatant was collected at each time point (D1, D4, and D7). Three wells for each surface modification were used and the experiment was repeated three times. The supernatant was centrifuged to remove cells and stored under −80 °C until further analysis. MultiPlex Biolegend kit was used as per the manufacturer's instructions to analyze the secreted cytokines from macrophages. Finally, flow cytometry (BD Fortessa X‐20) was used to obtain the expression of cytokines, and the data was analyzed using BioLegend's LEGENDplex data analysis software. However, it was difficult to assess all cytokines investigated (13 cytokines) because the concentration level of most of the cytokines measured was lower than the lowest detectable concentration. This is due to the use of unstimulated macrophages, which was the objective of our study.

##### Gene Expression

Gene expression of IL‐1β (pro‐inflammatory) and IL‐10 (anti‐inflammatory) from macrophages seeded on modified surfaces was performed for each time point (D1, D4, and D7). Three wells for each surface modification (6‐well plate) were used and the experiment was repeated three times. mRNA from macrophages seeded on modified surfaces was isolated at each point using PureLink RNA Mini Kit–RNA‐extraction kit (Thermo Fischer Scientific) as per the manufacturer's instruction. After isolation, the purity and concentration of mRNA was obtained from a nanodrop Lite Spectrophotometer (Thermo Fischer Scientific) and was stored at −80 °C until further analysis. cDNA synthesis was performed using isolated mRNA from each surface using iScript cDNA Synthesis Kit (BIO‐RAD) as per the manufacturer's instructions. Primers for genes were designed using the NCBI Primer‐BLAST tool (**Table** **1**). Reverse transcription‐polymerase chain reaction (RT‐PCR) was carried out using SsoAdvanced Universal SYBR Green Supermix (Bio‐Rad) as per the manufacturer's instructions. The data was analyzed using the RT‐PCR system (CFX Connect Real‐Time PCR system, BIO‐RAD).

**Table 1:** Primer sequence for gene expressions

Gene	Forward primer	Reverse primer
GAPDH	ATGTCGTGGAGTCTACTGGC	TGACCTTGCCCACAGCCTTG
IL‐1β	ATGCACCTGTACGATCACTG	ACAAAGGACATGGAGAACACC
IL‐10	CGCATGTGAACTCCCTGG	TAGATGCCTTTCTCTTGGAGC

##### Statistical Analysis

Each experiment was conducted with three parallel samples and repeated three times. Mean ± standard error mean (SEM) was obtained to perform statistical analysis using GraphPad Prism 6 (Graphpad, CA, USA). One‐way analysis of variance followed by Dunnett's multiple comparisons was performed for modified surfaces compared to smooth pOX surface for each time point (D1, D4, and D7). The significance interval was set as 0.05. Asterisks denote the significance level: *(0.05), **(0.01), ***(0.001), and ****(0.0001).

## Conflict of Interest

The authors declare no conflict of interest.

## Author Contributions

The manuscript was written through the contributions of all authors. All authors have given approval to the final version of the manuscript.

## Supporting information

Supplementary Material

## Data Availability

The data that support the findings of this study are available from the corresponding author upon reasonable request.
