# Supplementary material for: Nano‐Roughness‐Mediated Macrophage Polarization for Desired Host Immune Response
Source: Small Sci. 2023 Aug 13;3(10):2300080. doi: 10.1002/smsc.202300080 (PMC11935885; doi:10.1002/smsc.202300080)
Supplement: Supplementary file 1 — Supplementary Material [file SMSC-3-2300080-s001.pdf]

Supporting Information

Nano-Roughness-Mediated Macrophage Polarization for Desired Host Immune Response

Panthihage Ruvini L. Dabare[1#], Akash Bachhuka[2#*], Jing Yang Quek[1], Lluis F. Marsal[2], John Hayball[3] and Krasimir Vasilev[1,4*]

[1] UniSA STEM, University of South Australia, Mawson Lakes, South Australia, Australia, 5095,

[2] Department of Electronics, Electric, and Automatic Engineering, Rovira I Virgili University (URV), Tarragona, Spain 43007.

[3] Experimental Therapeutics Laboratory, UniSA Clinical and Health Sciences. University of South Australia, City East Campus, Adelaide, 5000, Australia

[4] College of Medicine and Public Health, Flinders University, Sturt Road, Bedford Park South Australia, 5042, Australia

Supporting Table 1: The comparison of Au content before and after overcoating

Surface	Amount of Au before overcoating (% At)	Amount of gold after overcoating (% At)
**16 nm**	0.12	0.07
**38 nm**	0.68	0.44
**68 nm**	1.58	1.02

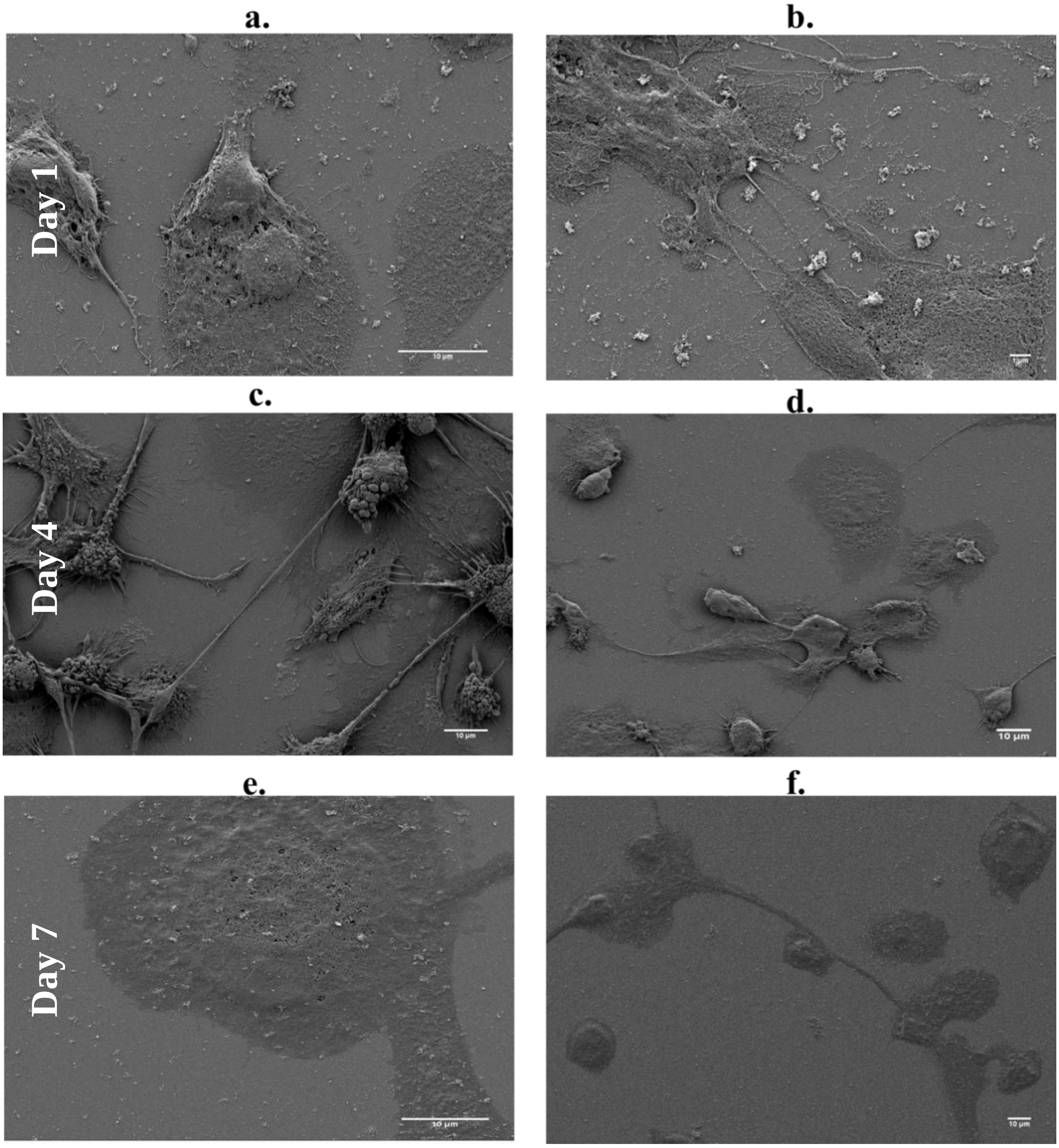

Figure S1: Macrophages on pOX surface with extended cytoplasm on D1 (a), Cell-cell interaction via spindle in D1 (b), further maintaining cell-cell interaction at D4 (c), larger pancake-like cells at D4 (d) firmly attached enlarged cells at D7 (e) and cell-cell interaction at D7 (f)

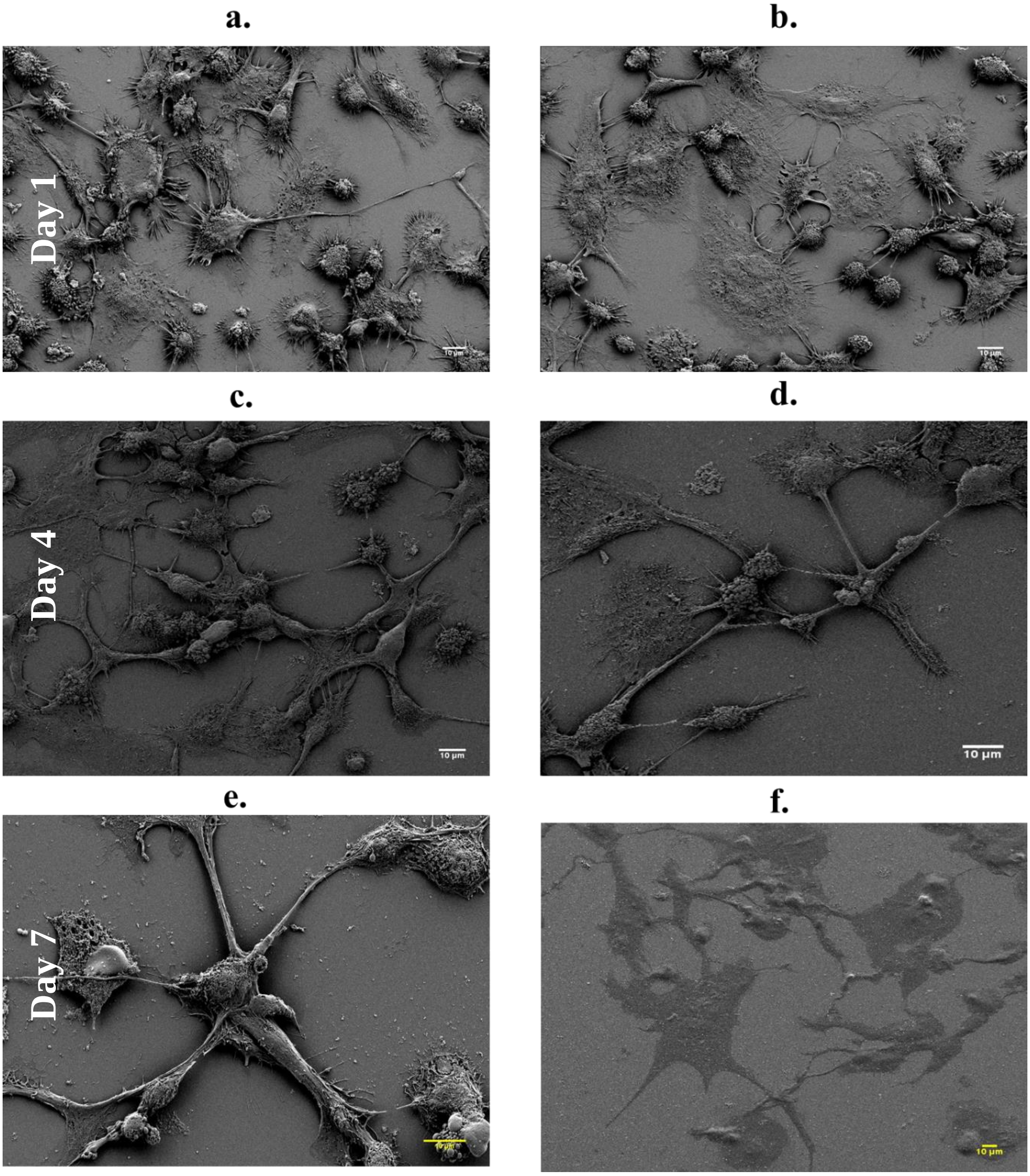

Figure S2: Macrophages on 16 pOX surfaces: with round cells on D1 (a) multi-nucleated cells at D1 (b), round cells and cells with extended cytoplasm at D4(c), cell-cell interaction through spindle at D4 (d) the way of cell interaction via filopodia at D7 (e) firmly attached cells at D7 (f)

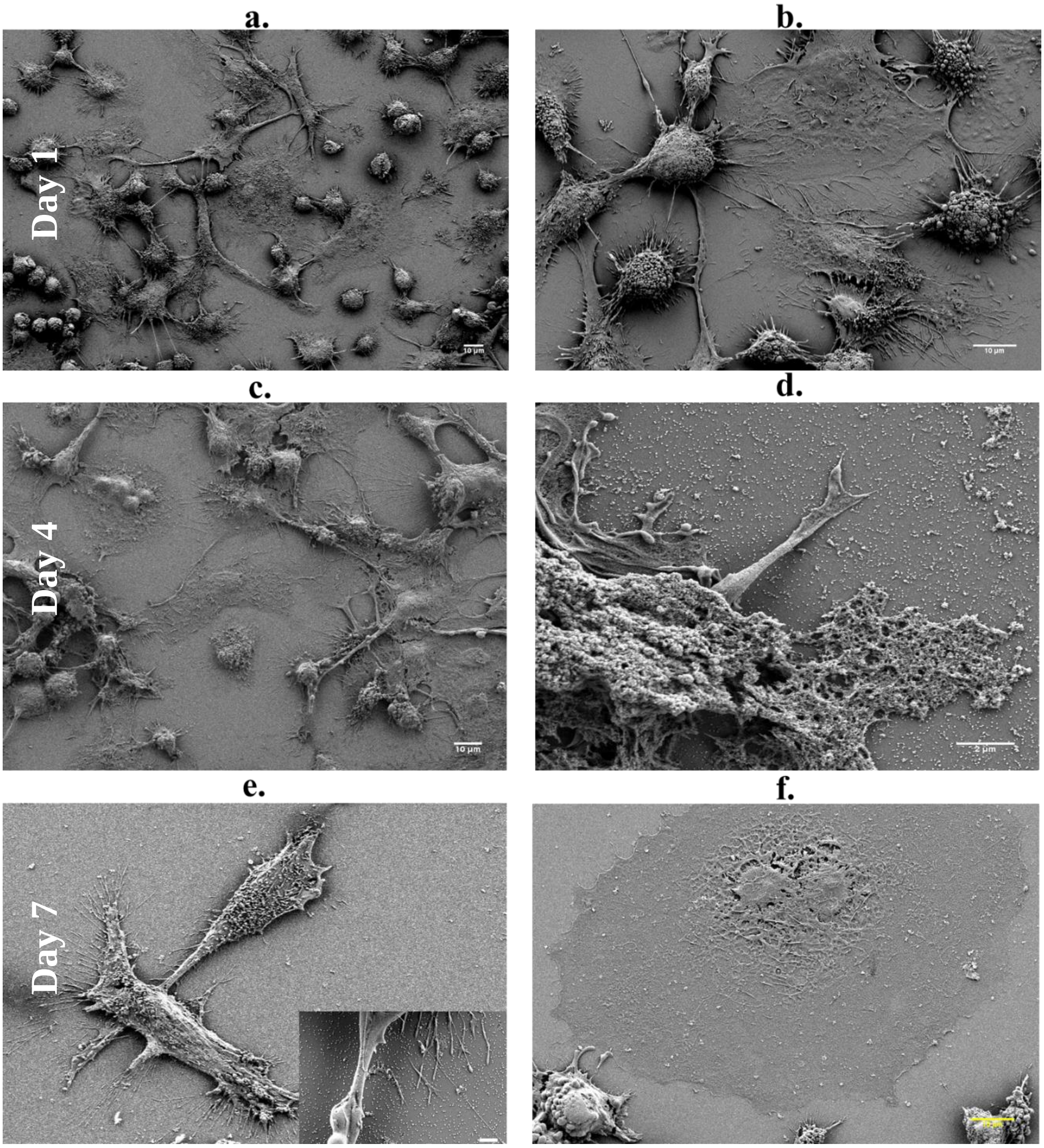

Figure S3: Macrophages on 38 pOX surfaces with round cells, elongated cells, and bipolar spindle cells on D1 (a) cell-cell interaction and pancake-like cells at D1 (b), multi-nucleated cells at D4(c), cell-surface interaction at D4 (d), elongated cells and spindle of a cell at D7 (e) firmly attached multi-nucleated cells at D7 (f)

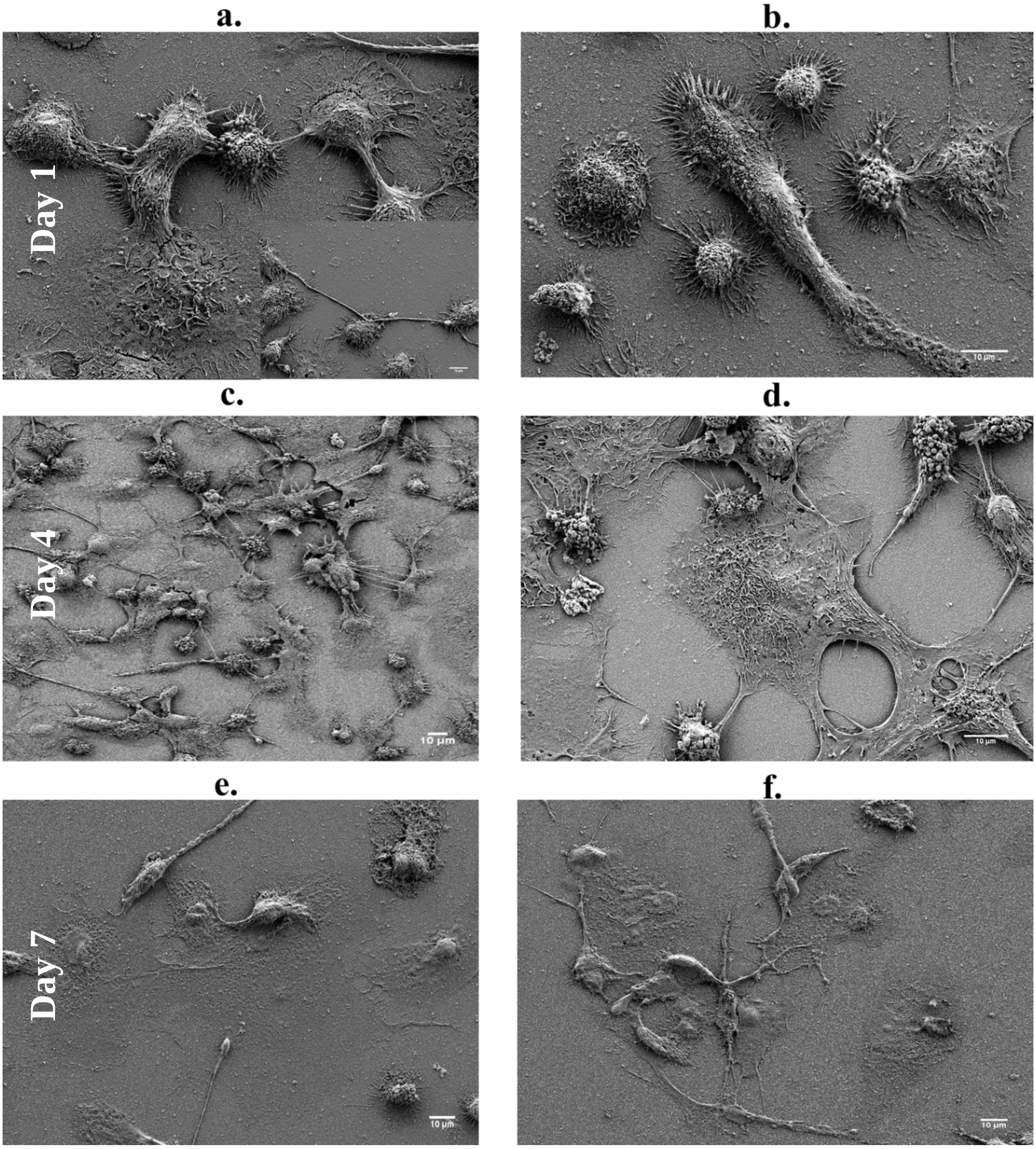

Figure S4: Macrophages on 68 pOX surface with round cells, pancake-like cells and cell-cell interaction on D1 (a) elongated cell and round cells at D1 (b), different types of morphologies at D4(c,d), highly spread cells at D7 (e) firmly attached multi-nucleated cells at D7 (f)
